# How achievement of a stroke care pathway is associated with functional outcome after stroke: a national, register-based study

**DOI:** 10.1186/s12913-025-12979-0

**Published:** 2025-07-01

**Authors:** Elin Bergh, Torunn Askim, Ole Morten Rønning, Stian Lydersen, Hild Fjaertoft, Bente Thommessen

**Affiliations:** 1 Department of Neuromedicine and Movement Science, NTNU, Trondheim, Norway; 2 Department of Neurology, Akershus University Hospital, Lørenskog, Norway; 3 Department of Mental Health, Regional Centre for Child and Youth– Mental health and Child Welfare (RKBU), NTNU, Trondheim, Norway; 4 The Norwegian Stroke Registry, St. Olavs Hospital, Trondheim, Norway

**Keywords:** Stroke, Stroke care pathway, Goal achievement, Acute stroke treatment, Treatment delay, Time use

## Abstract

**Background:**

Care pathways are introduced to ensure adherence to treatment guidelines for several patient groups. In the present study, we aimed to evaluate whether the achievement of key objectives within the recently implemented Norwegian stroke care pathway (SCP) was associated with functional outcome 3 months after stroke and to identify the characteristics of patients who achieved the recommended goals.

**Methods:**

We conducted a register-based study with data from the Norwegian Stroke Registry (NSR). Patients who were registered with acute stroke in 2019, one year after the SCP was introduced, were included. The functional outcome at 3 months was compared between ‘achievement’ and ‘non-achievement’ patients. The modified Rankin scale (mRS) was used to measure functional outcome at 3 months post-stroke, functional independence was defined as an mRS score of 0–2.

**Results:**

Among 2730 patients without missing data on achievement criteria, 272 (10%) were in the achievement group. The probability of being independent at 3 months post-stroke was significantly greater in the achievement group than in the non-achievement group (odds ratio (OR) 1.35, 95% CI 1.01 to 1.79, *p* = 0.043). However, subsequent sensitivity analyses did not replicate these findings, showing an average proportion of achievers of 7.2% (average odds ratios 1.12, 95% CI 0.88 to 1.45, *p* > 0.05). “Achievers” had better pre-stroke function, less likelihood of living alone, more severe strokes upon admission, and in addition, higher thrombolysis rates compared to “non-achievers.”

**Conclusion:**

We concluded that in the early phase after the SCP was implemented, between 7% and 10% reached goal achievement. The estimated association between achievement and independence 3 months post-stroke was significant in the primary analysis, but lost significance in sensitivity analyses.

## Introduction

Stroke is the third leading cause of mortality and the fourth most common contributor to Disability-Adjusted Life Years (DALYs) worldwide [1]. Over the past few decades, revascularization therapies—intravenous thrombolysis and thrombectomy—have significantly improved acute stroke management [2, 3]. As these treatments are highly time-sensitive, international guidelines recommend fast recognition and treatment of acute stroke [4]. One method of adhering to these guidelines while also developing proper and high-quality treatment has been to implement standardized care pathways. These pathways are defined as structured, multidisciplinary plans that outline the optimal sequence and timing of interventions, assessments, and treatments for patients with acute stroke. Their design is based on the best available evidence and clinical guidelines, and their aim is to ensure consistent, high-quality care, improve patient outcomes, enhance efficiency, and reduce variability in treatment across different healthcare providers [5].

In Norway approximately 10,000 patients are hospitalized with acute stroke each year [6]. Despite the establishment of national guidelines for acute stroke treatment in 2010, disparities in care seemed to exist across different regions and hospitals, which is one of the main reasons why Norwegian health authorities introduced a national standardized pathway of stroke care in 2018 [7–9]. The pathway is divided into two phases in accordance with key national quality indicators. Phase one covers the pre - and -intrahospital pathway and its time-specific goals are shown in Table 1.

Research has demonstrated that even small reductions in delay to stroke treatment may significantly affect outcomes. One minute of delay to treatment is shown to provide 1.8 extra days of healthy life in those receiving thrombolysis and 4.2 days in those receiving endovascular therapy [10–12]. Even for patients who do not receive reperfusion therapy, early arrival at the hospital is associated with a favourable outcome [13, 14]. Treatment within a comprehensive stroke unit increases the likelihood of survival and enhances the probability of being independent and living at home one year after stroke. Additionally, early admission to a stroke unit may further enhance these outcomes [15, 16].

**Table 1:**
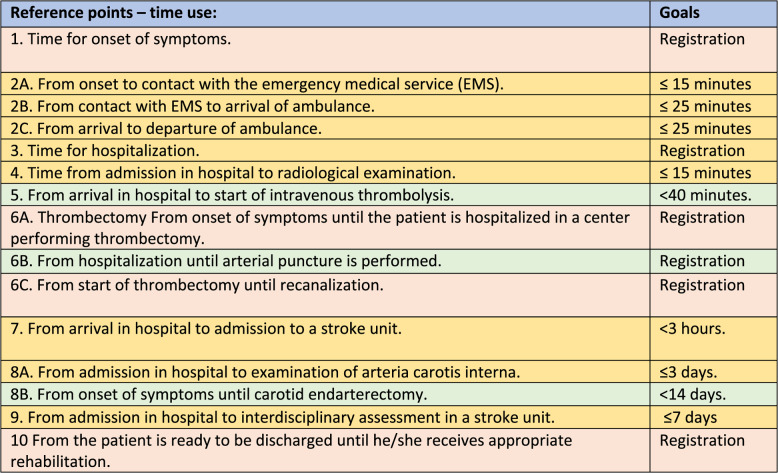
The stroke care pathway

Different colours indicate points of registration (pink), points with goals for time use (yellow), and points that only apply for a subgroup (green)

In a previous study, we evaluated the impact of the introduction of the SCP on functional outcomes after stroke [15]. No change in global function or mortality 3 months post-stroke was found. However, more patients were treated in a stroke unit, and more patients were discharged directly from the acute hospital stay after the introduction of the SCP [17].

Achieving pathway goals is an important measure of pathway effectiveness. Accordingly, we identified four essential goals that apply to all the patients, each of which was defined before conducting the analyses.

The primary aim of the present study was to determine whether achieving these four stroke care pathway goals—designed to reduce diagnostic and treatment delays—was associated with functional outcome three months post-stroke. Secondarily, we wanted to identify the characteristics of patients who achieved these goals.

We hypothesized that patients who achieved all four goals would have significantly better functional outcome at 3 months post-stroke than non-achievers.

## Methods

### Study design, setting and population

This register-based study uses data obtained in the acute phase and at the 3-month follow-up from the Norwegian Stroke Registry (NSR). Data regarding the stroke care pathway (SCP), including the goals with the target times, constitute a part of the NSR. The register is compulsory and all Norwegian acute care hospitals are obliged to report medical data on patients ⩾18 years admitted to hospital with acute stroke. Each hospital that registers data has designated contacts and registry administrators within the stroke department. Data for the registry are gathered from patient medical records during their hospital stay and at the 3-month follow-up through outpatient clinic visits, telephone interviews, or letters. The number of strokes registered in the NSR is calculated as the proportion of the total annual number of strokes reported to the Norwegian Institute of Public Health. The coverage rate of the register was 87% in 2019 [18].

All patients registered in the NSR with acute stroke in 2019, with a follow-up assessment 3 months post-stroke and without missing data were included in the present study.

### The goals of the stroke care pathway

The stroke care pathway includes goals related to the time elapsed from the onset of stroke symptoms until the patient is ready to be discharged from the hospital, as illustrated in Table 1. Three of the goals (1, 6 A and 10) are points of registration only. Goals 2B and 2 C—the time from contact with the EMS to arrival of an ambulance and the time from arrival to departure of the ambulance - are in large part covered by goal 3 - time from onset of symptoms to arrival at the hospital. Goal 4 measures the time from arriving at the hospital until CT/MRI is performed, and goal 7 measures the time from arrival at the hospital to admission to a stroke unit. Goals 5, 6 A, 6B and 6 C cover reperfusion therapy, and 8 A and 8B cover endarterectomy of carotid artery stenosis. These goals pertain only to subgroups and hence were not included in the present analyses. With respect to goal 9, i.e., the time for multidisciplinary assessment, the data were insufficient.

Therefore, the following goals of the SCP were used in our study:

(2 A) Time from symptom onset to contact with the emergency medical service (EMS) ≤ 15 min.
(3) Time from symptom onset to admission to the hospital ≤ 4 h.
(4) Time from arriving in the hospital until CT/MRI is performed *≤* 15 min.
(7) Time from arrival at the hospital to admission to a stroke unit ≤ 3 h.

“Achievement” was defined as fulfilling all 4 goals within the recommended times. “Non-achievement” was defined as fulfilling 0–3 of these goals.

All the included goals were selected prior to the analyses.

### Data collection

The data retrieved from the NSR comprise the following:

1. Baseline characteristics: age, sex, marital status, residence, and health region.
2. Cardiovascular risk factors: hypertension, atrial fibrillation, previous transient ischaemic attack (TIA), previous stroke, and diabetes mellitus.
3. Functional status: neurological deficit at admission measured by the National Institutes of Health Stroke Scale (NIHSS), and functional status before and three months post-stroke measured by the mRS [19].
4. Goals in the stroke care pathway: time for onset of symptoms, time for contact with emergency medical services (EMS), time for hospitalization, time for CT/MRI, and time for admission to a stroke unit.

### Outcomes

The primary outcome was good functional outcome (mRS 0–2) 3 months post-stroke. The mRS is a 7-point disability scale ranging from 0 to 6, where 0 corresponds to no symptoms and 5 corresponds to severe disability. A score of 6 denotes death. The scale is a widely used outcome measure in stroke trials, and multiple studies have demonstrated its validity [19, 20]. Evaluation of the mRS score prior to stroke was based on information from the patient, their next of kin and the patients’ medical records. For the secondary aim, the outcome was goal achievement.

### Statistics

Descriptive data are presented as counts and proportions for categorical variables and means and standard deviations (SDs) for continuous variables. The primary analyses were logistic regression with an mRS score of 0–2 (yes vs. no) at 3 months post-stroke as the outcome variable and achievement (yes vs. no) as the independent variable. We conducted analyses that were both unadjusted and adjusted one at a time and simultaneously for age, sex, living alone, NIHSS score at admission and pre-mRS score (0–2 versus 3–5). These variables were predefined as plausible confounders.

Logistic regression analyses with achievement of the pathway goals (yes or no, as the dependent variable) were used to identify the characteristics of patients who achieved the goals. In these analyses, age, sex, living alone, hypertension, diabetes mellitus, prior TIA, prior stroke, atrial fibrillation, etiology of stroke (ischemic or hemorrhagic), stroke severity (NIHSS) at admission, prestroke mRS (0–2 or 3–5) and health region were used as independent variables, one at a time. We present odds ratios (ORs) with 95% CIs for the main results.

To assess the robustness of our primary analyses, we conducted sensitivity analyses to address missing data on the four goals of the care pathway. Datasets were created imputing 0 (no) or 1 (yes) for those with one, two and three missing data points. We then repeated the logistic regression analyses for the primary aim with these datasets.

Statistical analyses were conducted via SPSS version 27 (IBM SPSS, Chicago, IL, USA).

## Results

**Fig. 1:**
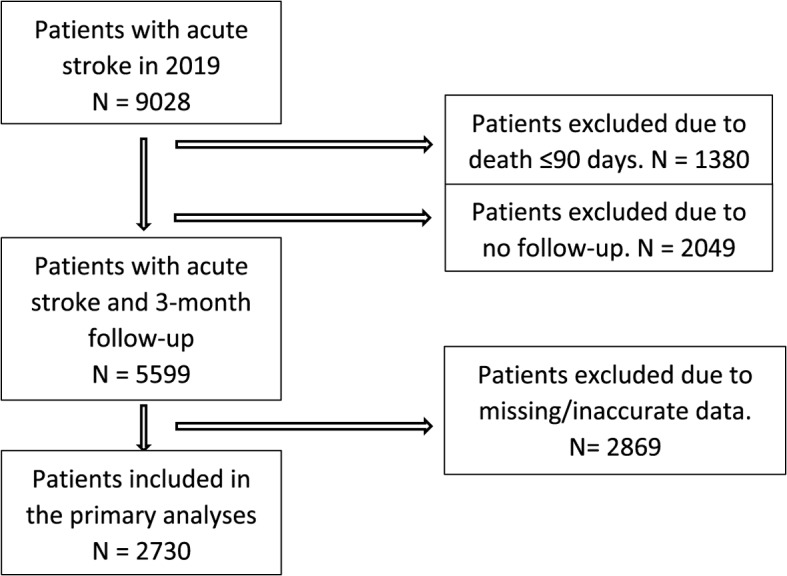
Flowchart showing inclusion/exclusion of patients

In total, 9028 patients with acute stroke were registered at the NSR in 2019. Of these, 5599 (62%) had a 3-month follow-up, and 2730 (30%) were included in our primary analyses. Figure 1 shows the flowchart illustrating the inclusion and exclusion of patients. The missing data were mostly due to missing registration of time for contact with the EMS.

In total, 272 (10%) of the 2730 patients achieved the four included target times. Contact with the EMS was the least fulfilled target, with only 20.4% contacting the EMS within 15 min. See Table 2 for missing data for the included goals.

**Table 2:** Number of patients with missing data on the four included goals

Missing data of included goals	*N*, %
None missing	2730 (48.8)
1 missing	1823 (32.6)
2 missing	858 (15.3)
3 missing	188 (3.4)
Total	5599

Table 3 shows the characteristics of the included patients in total and the two subgroups. Substantially more patients in the achievement group were living with someone prior to the stroke. Additionally, they had more severe stroke at admission and were to a greater degree independent (mRS 0–2) before the stroke, and the proportion receiving thrombolysis was more than twice as large.

**Table 3:** Patient characteristics in total and achievement and non-achievement groups

	Total *N* = 2730	Achievement group *N* = 272	Non-achievement group *N* = 2459
Demographics			
Age, *mean (SD)*,* y*	73.3 (12.3)	71.4 (13.4)	73.5 (12.1)
Female sex, *n (%)*	1169 (42.8)	115 (42.3)	1054 (42.9)
Living alone pre-stroke	1043 (38.4)	67 (24.8)	976 (39.9)
Risk factors			
Hypertension	1596 (58.6)	160 (58.8)	1436 (58.5)
Diabetes Mellitus	481 (17.7)	36 (13.3)	446 (18.2)
Previous TIA	272 (10.1)	25 (9.3)	247 (10.2)
Previous stroke	640 (23.5)	64 (23.6)	576 (23.5)
Previous atrial fibrillation	628 (23.1)	54 (19.9)	574 (23.4)
Ischemic stroke	2462 (90.6)	242 (89.0)	2221 (90.8)
Thrombolysis	804 (29.5)	172 (63.2)	633 (25.7)
Degree of disability			
NIHSS at admission, *mean*	4.9 (5.27)	6.8 (6.6)	4.7 (5.04)
mRS pre-stroke, *n (%)*			
-mRS 0–2	2263 (88.8)	238 (92.6)	2025 (88.4)
-mRS 3–5	284 (11.2)	19 (7.4)	265 (11.6)

The data are presented as numbers (%) unless otherwise indicated

*SD* Standard deviation, *TIA* Transient ischaemic attack, *NIHSS* National Institute of Health Stroke Scale, *mRS* Modified Rankin scale

At 3 months post-stroke the probability of having a good functional outcome was significantly greater in the achievement group than in the nonachievement group (OR 1.35, 95% CI 1.01 to 1.80, *p* = 0.043) and was even greater when adjusted for age, sex, living alone, pre-stroke mRS score and NIHSS score at admission (OR 1.82, 95% CI 1.24 to 2.67, *p* = 0.002). As shown in Table 4, the change in the OR was due mainly to adjusting for the NIHSS score at admission.

**Table 4:** Logistic regression with mRS 0–2/3–5 as the dependent variable and achievement in the stroke care pathway as the independent variable

Achievement of SCP	N	Odds ratio	95% CI	*p*-value
Unadjusted	2730	1.35	1.01 to 1.79	0.043
Adjusted separately for				
• Age	2730	1.24	0.92 to 1.68	0.15
• Female sex	2730	1.34	1.00 to 1.79	0.043
• Living alone	2717	1.26	0.94 to 1.68	0.123
• Pre-mRS 3–5	2546	1.24	0.89 to 1.72	0.19
• NIHSS at admission	2484	2.08	1.50 to 2.90	<0.001
Adjusted for all	2332	1.82	1.24 to 2.67	0.002

However, in subsequent sensitivity analyses imputing “no” or “yes” for missing data on pathway goals, the association between achieving goals of the SCP and independence (mRS 0–2) post-stroke was no longer significant, as shown in Table 5. These sensitivity analyses yielded lower ORs than did the primary analyses, with an average proportion of achievers of 7.2% and an average OR of 1.12, 95% CI of 0.88 to 1.455, *p* > 0,05.

**Table 5:** Sensitivity analyses

	Achievers	OR estimate	CI	*p* value
Data on all 4 criteria Complete case				
No imputation	272/2730 (10.0%)	1.35	1.01 to 1.79	0.043
Data on at least 3 criteria				
Impute 0	272/4553 (6.0%)	1.11	0.84 to 1.47	0.47
Impute 1	412/4553 (9.0%)	1.12	0.91 to 1.46	0.23
Data on at least 2 criteria				
Impute 0	272/5411 (5.0%)	1.07	0.81 to 1.42	0.64
Impute 1	472/5411 (8.7%)	1.15	0.93 to 1.44	0.21
Data on at least 1 criterion				
Impute 0	272/5599 (4.9%)	1.08	0.82 to 1.42	0.61
Impute 1	542/5599 (9.7%)	1.20	0.97 to 1.48	0.09

mRS 0–2 (yes vs. no) as the dependent variable and achievement (yes vs. no) as the independent variable with imputed datasets

In terms of the secondary aim, the unadjusted logistic regression model with achievement as an outcome revealed that achieving the goals of the pathway was associated with living with someone pre-stroke (OR 2.01, 95% CI 1.51 to 2.68, *p* < 0.001) and with more severe stroke at admittance to the hospital (OR 1.73, 95% CI 1.33 to 2.26, *p* < 0.001). Significant regional differences were found, and living in one of the regions was negatively associated with goal achievement compared with living in a reference region both in the main and in the subgroup analyses (OR 0.59, 95% CI 0.43 to 0.83; *p* = 0.002).

## Discussion

The primary analyses revealed that good functional outcome 3 months after acute stroke was significantly associated with good adherence to the four selected goals of the stroke care pathway. However, the sensitivity analysis did not support these findings. Data were evidently not missing completely at random. It cannot be ruled out that the association between SCP achievement and functional outcome was weak or entirely absent. The conflicting results must be interpreted with caution.

The pathway focuses on reducing time delay, which is vital for a good treatment outcome in acute stroke patients most importantly by facilitating revascularization treatment. The odds ratio (OR) of being independent at 3 months with thrombolysis compared with placebo decreases with treatment delay [9]. In our study, only 10% of the patients achieved the four chosen goals. Since two of the achievement goals involve early contact with EMS and early hospital admission, this suggests that the achievement group reaches the hospital sooner, which may explain the higher thrombolysis rate observed in this group compared to the non-achievement group, namely 63% versus 26%. This has most likely contributed significantly to the association with good outcomes in the adherence group.

Surprisingly, only one out of five of the patients with available data contacted the EMS within the recommended target time, which could be explained by a challenge in recognizing stroke symptoms [21–23]. Campaigns focusing on increasing stroke awareness in the public are effective, but the effect declines after campaign closure, implying that these campaigns need to be repeated [24]. Repeating the message across diverse media channels could be a tool to reach a broader audience. As acronyms can make stroke signs easier to remember, the campaigns could emphasize FAST (Face drooping, Arm weakness, Speech difficulty, Time to call emergency services). The importance of acting fast and contacting the emergency service should be reinforced in the campaigns. As most of the patients undergoing a stroke have risk factors, the high-risk groups should be targeted such as senior centers [25]. Yet, some studies show that there is a knowing-doing gap, meaning that knowing the symptoms does not always translate to action, this should be considered when new campaigns are being developed [26]. Intra-hospital delays can be reduced through effective stroke treatment protocols, streamlined emergency department processes, and quick access to imaging. In our study, only 59% of patients meeting target times were admitted to a stroke unit within three hours of arrival, underscoring the need for rapid ED management [27].

The characteristics of patients who achieved the goals of the SCP differed substantially from those with non-achievement. Most notably more patients in the achievement group were living with someone and were functional independent prior to the stroke. These differences may themselves lead to improved outcome regardless of adherence to the pathway. Additionally, the patients in the achievement-group had more severe stroke at admission.

This is in accordance with the findings of previous studies [25, 28–30]. Patients with a reduced functional status pre-stroke and especially if they are living alone, are less able to contact someone when they experience symptoms of acute stroke. Additionally, symptoms of comorbidities might complicate the examination and make it more difficult to recognize symptoms as acute strokes [21]. Our findings suggest that improving achievement may require targeted efforts for those who live alone with functional impairments.

The SCP is intended to reduce variations in stroke care throughout the country. Nevertheless, an important finding in our study was regional differences in the time from the onset of stroke to contact with EMS, from onset of stroke to hospitalization, and time from hospital admission to thrombolysis and admission to a stroke unit. These regional differences could originate from different organizations of acute stroke treatment, different regional procedures and routines, distances to hospitals and differences in populations living in urban and rural settings [22, 31]. Rapid and streamlined management in the emergency department is essential to improve in-hospital management, which may vary across hospitals. According to the NSR, hospitals with more than 50 patients receiving thrombolytic treatment per year are more likely to give tPA within 40 min [18]. Some hospitals have focused on reducing the time to thrombolysis by routinely performing simulation-based team training, which could be recommended for all hospitals treating patients with acute stroke. A recently published Norwegian study found that patients living in rural areas with long transport times to hospitals had a lower rate of reperfusion therapy. Both mobile stroke units and prehospital CT and thrombolytic centers are prehospital alternatives for diagnostics and treatment for people living in remote areas and that in some studies show promising results [32, 33].

However, further research is needed to deepen our understanding, identify the causes of these differences, and provide recommendations for improvement. Bringing attention to the disparities may help raise awareness of the variation in treatment practices across the country.

Regular awareness campaigns to increase the recognition of stroke symptoms, training program for EMS personnel to swiftly identify and transport suspected stroke patients to hospitals and optimize admission procedures for acute stroke patients are measures that may be implemented to further reduce unnecessary delays in hospitals and regions that need to improve their stroke care.

### Strengths and limitations

The large sample size and the high coverage rate of the NSR (87%) are strengths of the present study. However, using a register always includes a risk of inaccurate registration and incomplete and missing data. Excluding patients without a 3-month follow-up and patients with missing data left us with only 30% of the total population, which could introduce selection bias and cause an overestimation of the effect of the SCP. In our previous study, we compared the baseline characteristics of the included and excluded patients and reported that the non-follow-up cohort was significantly older, had poorer pre-stroke functioning, and was institutionalized to a greater extent than the group with a 3-month follow-up [17].

The discrepancy between the results of our primary analysis and the sensitivity analyses indicates a reduced robustness of our analyses. While the overall trend remained positive, caution is warranted when generalizing these results. Most likely, the registration will improve with time, thereby reducing the amount of missing data. Our conclusion should be interpreted with the understanding that the effect of the stroke care pathway may be sensitive to sample selection and data missingness, underscoring the need for further replication.

Owing to the lack of data, overlap of target times and two of the target times addressing only a minority of the population, we focused our analyses on four of the goals of the pathway. However, the selected goals are all significant in determining the outcome of stroke. Goals 2 A and 3 address the duration from symptom onset to contact with the EMS and the time to hospitalization. Prompt EMS communication is crucial for achieving early hospital arrival [23, 34]. Previous research has shown that each minute saved in thrombolysis leads to significant gains in disability-free life, highlighting the importance of rapid admission and treatment [10, 33]. Early CT examination without delay (Goal 4) to rule out hemorrhage before potential reperfusion treatment is a prerequisite for successful treatment. Goal 7 focuses on the duration from hospital arrival to admission to a stroke unit. A delay in stroke unit admission has been linked to prolonged hospital stays and poorer functional outcomes [33].

However, it is important to keep in mind that many factors may affect outcome after stroke in addition to the goals we have analysed and those confounders that we have adjusted for. The investigation, treatment and rehabilitation of patients, in addition to their cognitive function, socioeconomic status and educational level, are all associated with outcomes after stroke. The approach with a binary definition of “achievement” versus “non-achievement” might have simplified the complexity of stroke care, but since the study aimed to assess the impact of the SCP and identify the characteristics of patients who achieved the pre-defined goals of the pathway, we deemed it most appropriate to categorize the patients into two groups.

It is well documented that changing clinical practice takes time, and the fact that only 10% of the included patients achieved the goals suggests that the SCP was not fully implemented. Hence, it might be too early to identify the full effect of the pathway, especially with respect to functional outcome. However, further implementation of the SCP and attention to the regional differences revealed are expected to increase the focus on reducing the time delay.

Additionally, it is plausible that the observed achievements in target time could also be attributed to a general change in clinical practice in addition to the implementation of the SCP, as national guidelines have emphasized the importance of prompt treatment during the acute phase since 2010.

## Conclusion

This study revealed that less than 10% of stroke patients reached achievement of the recommended target times one year after the introduction of the stroke care pathway in Norway. The “achievers” were to a greater degree independent and living with someone pre-stroke and had more severe strokes at admission to hospital.

The significant association between SCP and good functional outcome 3 months post-stroke observed in the primary analyses, was not robust and lost significance in the sensitivity analysis, which could mean that the observed association was weak or even absent.

Few patients contacted the emergency medical services within the recommended time. This is a critical factor in reducing time delays and warrants greater emphasis. Additionally potential remains to improve intrahospital measures by minimizing delays to imaging and stroke unit admission. The uncovered regional differences show inconsistency in treatment nationwide.

Hopefully, future studies will demonstrate improved implementation of the SCP and associated benefits of adherence as a quality improvement measure, ultimately enhancing and optimizing stroke services.

## Data Availability

The data used in this study are from the Norwegian Stroke Registry. The data are anonymized and does not include any identifiers. The data were available for the authors after application, but to protect the participants they are not publicly available. See: https://helsedata.no/en/forvaltere/norwegian-institute-of-public-health/norwegian-stroke-registry/.
